# Enhanced Solubilisation of Six PAHs by Three Synthetic Cyclodextrins for Remediation Applications: Molecular Modelling of the Inclusion Complexes

**DOI:** 10.1371/journal.pone.0044137

**Published:** 2012-09-19

**Authors:** Esmeralda Morillo, María Antonia Sánchez-Trujillo, José Ramón Moyano, Jaime Villaverde, María Eulalia Gómez-Pantoja, José Ignacio Pérez-Martínez

**Affiliations:** 1 Institute of Natural Resources and Agrobiology of Seville, Agrochemistry and Soil Conservaton Department, Science Research Council (IRNAS-CSIC), Seville, Spain; 2 Department of Pharmacy and Pharmaceutical Technology, Faculty of Pharmacy, University of Seville, Seville, Spain; University of Kansas, United States of America

## Abstract

Solubilisation of six polycyclic aromatic hydrocarbons (PAHs) (acenaphthene, anthracene, fluoranthene, fluorene, phenanthrene and pyrene) by three synthetic cyclodextrins (CDs) (2-hydroxypropyl-β-CD, hydroxypropyl-γ-CD and ramdomly methylated-β-CD) was investigated in order to select the CD which presents the greatest increase in solubility and better complexation parameters for its use in contaminated scenarios. The presence of the three cyclodextrins greatly enhanced the apparent water solubility of all the PAHs through the formation of inclusion complexes of 1∶1 stoichiometry. Anthracene, fluoranthene, fluorene and phenanthrene clearly presented a higher solubility when β-CD derivatives were used, and especially the complexes with the ramdomly methylated-β-CD were favoured. On the contrary, pyrene presented its best solubility results when using 2-hydroxypropyl-γ-CD, but for acenaphthene the use of any of the three CDs gave the same results. Complementary to experimental phase-solubility studies, a more in-depth estimation of the inclusion process for the different complexes was carried out using molecular modelling in order to find a correlation between the degree of solubilisation and the fit of PAH molecules within the cavity of the different CDs and to know the predominant driving forces of the complexation.

## Introduction

Polycyclic aromatic hydrocarbons (PAHs) have received considerable attention as environmental organic pollutants because some of these compounds are highly carcinogenic or mutagenic. They are widely present in the air, water, soil and sediments, and belong to the so called “persistent organic pollutants” (POPs) which are defined as a set of organic compounds that: (I) possess toxic characteristics; (II) are persistent; (III) are liable to bioaccumulate; (IV) are prone to long-range atmospheric transport and deposition; (V) can result in adverse environmental and human health effects at locations near and far from their sources [1].

PAHs are the product of any incomplete combustion process involving material containing C and H (e.g. coal, oil, petrol, wood) [2]. Although some PAHs in the environment arise from natural combustion, emissions from anthropogenic activities predominate [3], [4]. They are adsorbed strongly to the organic fraction of sediments and soils [5]. As a consequence, PAHs degradation in soil is slow, and bioavailability can decline during ageing, becoming persistent contaminants. Their aqueous solubility is very low and this is the most important limiting factor in the clean-up of PAH-contaminated sites. To improve their desorption efficiency from soils, mobility and bioavailability various extracting agents have been used, such as cosolvents and surfactants, but they have some disadvantages: to be toxic to humans, to harm resident microbial population, or to form high-viscosity emulsions difficult to remove due to their low water-solubility [6].

Cyclodextrins (CDs) have been proposed as an alternative agent to enhance water solubility of hydrophobic compounds [7], [8]. CDs are cyclic oligosaccharides, containing 6 (α-CD), 7 (β-CD) or 8 (γ-CD) α-(1,4)-linked glucose units, formed from enzymatic degradation of starch by bacteria. The most important structural feature of these compounds is their toroidal shape, with hydrophobic interior cavity and hydrophilic shell [9]. They are capable of forming inclusion complexes both in solution and in solid state with a variety of guest molecules, which are placed in their hydrophobic interior cavity. CDs are widely used in pharmaceutical science [10], [11], but recently CDs have aroused considerable attention in many other fields, such as, nanocomposite technologies, chromatography, biotechnology, or agriculture [12], due to the low-cost productions of some of them. CDs have been used also in environmental applications to improve the remediation of contaminated soil, since they have the ability to increase the apparent water solubilities of low-polarity organic compounds, reducing their sorption and facilitating their mobilization [13]–[16]. This transfer of the contaminant from the soil into soil solution is an important way of improving the biodegradable fraction of POPs in soils [17], and in particular of PAHs [18]–[20].

The determination of the apparent water solubilities of PAHs in CDs solutions is crucial to select the most proper CD for solubilization of a wide variety of PAHs. However, normally the published papers only deal with the study of one or two PAHs, being phenanthrene and naphtalene the most frequently used. Moreover, the results obtained when PAH solubilities are measured vary depending on the methodology used [21], and, therefore, it is difficult to compare levels of PAH solubility influenced by the presence of CDs reported by different authors.

However, when a soil has to be decontaminated it is necessary to have a comparative view of the effect of several CDs on the solubility of a wide range of PAHs. Therefore, the aim of the present paper was to study and compare the influence of three different CDs (2-hydroxypropyl-β-CD, HP-β-CD; hydroxypropyl-γ-CD, HP-γ-CD; and randomly methylated-β-CD, RAMEB) on the solubility of six PAHs (selected among those 16 proposed by the US-EPA as the most frequently-occurring and/or dangerous in environmental samples), in order to select the CD which in general presents the best solubility results. HP-β-CD was selected due to its demonstrated ability to mimic the bioavailable concentrations of soil-associated nonpolar organic contaminants; RAMEB, in spite of being also a β-CD derivative, presents some different characteristics in comparison to HP-β-CD, and has been also used for soil decontamination; HP-γ-CD presents a higher hydrophobic cavity which could give advantage in the case of complexation for some voluminous PAHs.

Interactions between PAH and CDs were also studied using in silico molecular modelling. After a conformational analysis of monomers, a search of minimum energy structures was carried out at molecular mechanics level (MM), in order to find a correlation between the degree of solubilisation and the fit of PAH molecule within the cavity of the different CDs and to know the predominant driving forces of the complexation, together with energy changes and stability of inclusion complexes.

## Materials and Methods

### Materials

**Figure 1:**
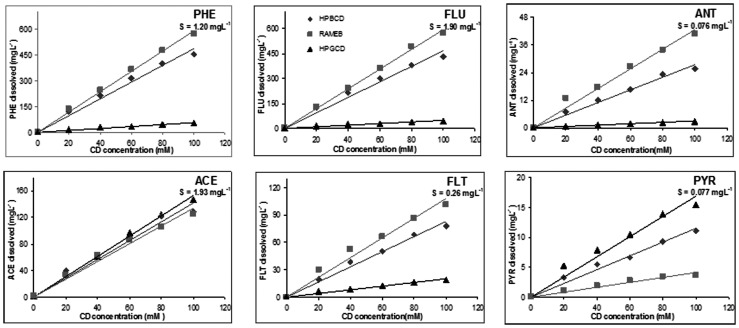
PAHs structures and phase solubility diagrams. In the presence of HP-β-CD (♦), RAMEB (▪) and HP-γ-CD (▴).

Powdered PAHs (acenaphthene, ACE, anthracene, ANT, fluoranthene, FLT, fluorene, FLU, phenanthrene, PHE, and pyrene, PYR) and PAHs stock solutions in methanol (purity >98%) were purchased from Sigma-Aldrich (Madrid, Spain). The molecular structures of the PAHs used are shown in Figure 1.

2-Hydroxypropyl-β-CD (HP-β-CD), randomly methylated-β-CD (RAMEB) and hydroxypropyl-γ-CD (HP-γ-CD) were supplied by Cyclolab (Budapest, Hungary). The average degree of substitution per CD ring were equal to 3, 12, and 4.5, respectively.

### Solubility studies

Solubility studies were performed according to Higuchi and Connors method [22]. 5 mg of each PAH were added to aqueous solutions (10 mL) containing increasing dissolved concentrations (from 0 to 0.1 M) of each CD. The experiments were carried out in triplicate. Flasks were sealed and shaken at 25°C for 1 week. The concentration of each PAH in solution was determined using a Shimadzu HPLC chromatograph equipped with a chromatographic column, Kromasil 100 C18 reverse-phase (5 µm, 15×0.4), coupled to a fluorescence detector (Shimadzu RF-10APXL). The chromatographic conditions were as follows: mobile phase, acetonitrile/water (60∶40); flow, 1.5 mL min^−1^; temperature, 30°C. For fluorimetric detection of PAHs the excitation/emission wavelengths pairs (nm) used were: ACE, 280/330; ANT, 240/450; FLT and PYR, 240/410; FLU, 280/330; PHE 250/365. Analysis of 1∶1 (v/v) hexane extract of the aqueous supernatant solutions was performed (PAHs were completely recovered in the separated hexane phase due to their much higher solubility in hexane than in CD solutions), and the calibration curve was also prepared in hexane. To obtain the phase solubility diagrams, the concentration of the PAHs dissolved versus the increasing concentrations of the CDs used were represented.

The apparent stability constants Kc for those inclusion complexes whose stoichiometry was 1∶1 were calculated from the slope of the straight lines obtained in the phase solubility diagrams type A_L_, following the equation proposed by Higuchi and Connors [22]:
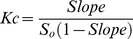
(1)S_0_ is the PAH equilibrium concentration in the absence of CDs; Slope is the slope of the phase solubility diagram.

Another parameter that can be obtained from the solubility diagram is the Solubilization Efficiency (Se), defined as the increment of the apparent solubility of the PAH at a fixed concentration of CD (S_CD_) of 100 mM with respect to the PAH solubility in the absence of CD (S_0_):
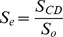
(2)


### Molecular modelling

Molecular modelling studies were carried out by Hyperchem software package, version 8.0.8 [23]. Structure of RAMEB was obtained from Protein Data Base (PDB) (PDB ID: 2QKH) and built by addition of methyl substituents, in order to reach the substitution degree (DS∼12). HP-β-CD structure was built by addition of 2-hydroxypropyl substituents to a crystal structure of β-CD, obtained from PDB (PDB ID: 3CGT). In the same manner, HP-γ-CD was built from the γ-CD crystalline structure (PDB ID: 1VFU). CD structures were built under the indications of Cyclodextrin Knowledge Base [24]. PAHs molecular structures were drawn in Hyperchem.

In order to find the most stable conformation both for PAH and CDs, a conformational analysis was performed by using the Conformational Search program included in Hyperchem. The following conditions were set up for conformational search: variation of the flexible torsion angles ±60° ÷ ±180°, energy criterion for acceptance of the conformation 4 Kcal/mol (8.148 KJ/mol) above minimum, all conformations with atomic distances lower than 0.5 Å and differences between torsion angles lower than 15° were not considered, as well as conformations with energy differences lower than 0.05 Kcal/mol (0.2092 KJ/mol) (duplicates); the maximum number of optimization and iterative calculations was 1000 and maximum 100 conformations were retained. The hydrogen atoms were neglected [25]. The treatment was carried by the MM+ force field included in Hyperchem, with a root mean square (rms) convergence of 0.01 Kcal/Å mol (0.04184 KJ/Åmol). MM+ approach is good enough to explain the formation of CD inclusion complexes [26], [27].

In order to identify the structure of the complexes corresponding to the global minima on the potential surface energy of CD, our studies were carried out according to the method previously described in the literature [28], [29]. Briefly, CD molecule was oriented until its glycosidic oxygen atoms were placed onto the XY plane, being the center considered as the center of the coordination system. The secondary hydroxyl groups of CD were oriented towards the positive direction of the orthogonal Z-axis. Then, the guest molecule was placed along the Z axis of the coordination system (from −10 to +10 Å), taking into consideration different orientations of the guest molecule if the CD complex has several possible binding modes, as occurs with FLT and PYR.

In a third step, the guest molecule is allowed to enter and then pass through the CD molecule by steps. At each step of 1 Å, a full geometry optimization procedure was developed in vacuum by the MM+ force field (Polak-Ribiere conjugate gradient algorithm with a rms convergence of 0.01 Kcal/Å mol (0.04184 KJ/Åmol)). Due to the asymmetrical shape of minimized CD, PAH molecules were slightly rotated in order to prevent close contacts between PAH and CD groups at the starting resultant structures generated at each translation step. Results are presented as energetic outcomes expressed as complexation, interaction and deformation energies (equations 3 to 5). The complexation energy (eq 3) is the difference between the energy of the complex and the sum of the energy of each monomer at their respective equilibrium geometry. The interaction energy (eq 4) is defined as the difference between the energy of the complex and the sum of the energies of both partners at their complex geometry. The deformation energy (eq 5) is determined by the difference between the energy of one of the partners of the complex and its energy at the complex geometry [30].
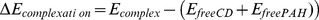
(3)

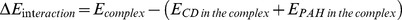
(4)

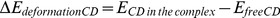
(5)E_CD in the complex_ is the single point energy value of CD in the optimized complex [31]–[34].

## Results and Discussion

The phase solubility diagrams of the PAHs selected in the presence of different concentrations of the CDs are shown in Figure 1. A linear increase in the solubility of all PAHs is observed with increasing concentrations of CDs, indicating an A_L_ classification according to Higuchi and Connors [22], since a solubility limit is not obtained in the range of CDs concentrations used and only dissolved complex is formed in the phase solubility isotherm. All the diagrams are straight lines, in general with coefficients of determination (R^2^) greater than 0.99, and slope <1, ascribed to the formation of complexes with 1∶1 stoichiometry. The apparent formation constants (Kc) and the solubility efficiency (Se) of these CDs are shown in Table 1.

**Table 1:** Apparent stability constants (Kc, M^−1^) and solubilization efficiency (Se) for the PAH-CD complexes.

	HP-β-CD			RAMEB			HP-γ-CD		
	Se	Kc	R^2^	Se	Kc	R^2^	Se	Kc	R^2^
**ACE**	**66** (±20)	**661** (±35)	**0,9971**	**65** (±2.0)	**644** (±12)	**0,9944**	**76** (±13)	**758** (±41)	**0,9742**
**ANT**	**336** (±21)	**3284** (±204)	**0,9961**	**535** (±9.8)	**5399** (±52)	**0,9917**	**36** (±3.8)	**234** (±35)	**0,9794**
**FLU**	**227** (±30)	**2207** (±380)	**0,9990**	**302** (±30)	**2906** (±164)	**0.9751**	**23** (±1.9)	**218** (±14)	**0,9903**
**FLT**	**300** (±37)	**2967** (±130)	**0,9876**	**390** (±8.4)	**3909** (±30)	**0,9679**	**73** (±3.1)	**701** (±30)	**0,9942**
**PHE**	**376** (±50)	**3871** (**±**322)	**0,9995**	**477** (±54)	**4925** (±511)	**0,9884**	**45** (±2.1)	**447** (±17)	**0,9991**
**PYR**	**144** (±8.6)	**1312** (±42)	**0,9920**	**48** (±2.1)	**525** (±23)	**0,9942**	**201** (±28)	**1838** (±252)	**0,9944**

In the case of complexation with HP-β-CD, the comparative study of Kc shows a great difference among PAHs, being almost 6-fold higher the Kc value for PHE than that for ACE. The ability of a CD to form an inclusion complex with a guest molecule is function of two key factors. The first is steric and depends on the relative size of the CD to the size of the guest molecule, or certain key functional groups within the guest. The second is the thermodynamic interactions between the different components of the system (CD, guest, solvent) [35].

The solubility efficiency (Se) of HP-β-CD towards the PAHs shows the same sequence as the corresponding Kc values, which are shown in Table 2, and it also corroborates the preceding deduction. It is important to emphasize the great difference in solubility of the inclusion complexes with PHE, ANT and FLT in the presence of HP-β-CD, increasing up to 300-fold (Table 1). Wang and Brusseau [36] and Fenyvesi et al. [37] observed that HP-β-CD was especially good solubiliser of soil contaminants, improving their solubility with 1–3 orders of magnitude depending on the size and shape of the solute. The PAHs that showed a lower increase in solubility were PYR and ACE, but even those are appreciable (144 and 66-fold higher, respectively).

**Table 2:** Decreasing order of Se, Kc and PAHs apparent solubility* (reached with 0.1 M of the CDs).

	Solubility	Se and Kc
**HP-β-CD**	PHE≥FLU>ACE>FLT>ANT>PYR	PHE>ANT>FLT>FLU>PYR>ACE
**RAMEB**	FLU≥PHE>ACE>FLT>ANT>PYR	ANT>PHE>FLT>FLU>ACE>PYR
**HP-γ-CD**	ACE>PHE>FLU>FLT>PYR>ANT	PYR>ACE>FLT>PHE>ANT>FLU

* PAHs aqueous solubility (S) (mg l^−1^): ACE (1.93)≥FLU (1.90)>PHE (1.20)>>FLT (0.26)>>PYR (0.077)≥ANT (0.076).

The different values of Kc and Se indicate that PAHs solubilisation by CDs depends on their respective molecular structures. Since the size of the different CD molecules is fixed, only those compounds with a molecular size similar or smaller and an appropriate structure have options to be included inside of CDs internal cavities. According to Wenz et al. [38], the major driving forces of the formation of CD inclusion compounds are hydrophobic and van der Waals interactions between the inner surface of the CD ring and the hydrophobic sites on the guest. The higher the binding constant, Kc, the better a guest fills out the CD cavity. In this sense, the results presented in Table 1 indicate that the best molecular accommodation in the cavity of HP-β-CD was presented by PHE and ANT, due probably to their adequate size and shape and, therefore, with a strong interaction with the active centres in the cavity.

The parameters relative to the dimensions of the six PAHs selected in this study are shown in Table 3. Taking into account that the cavity of β-CD and their derivatives present a minimum internal diameter about 5.8–6.5 Å and a depth of 7.9 Å [31], [38], the PAH that presents the more similar lower width is PHE (5.50 Å, Figure 1), that is the PAH which presented the highest increase in Se and Kc values when formed inclusion complex with HP-β-CD.

**Table 3:** Dimensions (in Å) of the PAHs studied measured in Hyperchem 8.0.8.

	Width (Å)	Length (Å)
**ACE**	5.89	6.74
**ANT**	4.91	9.14
**FLT**	6.71	8.52
**FLU**	5.23	8.97
**PHE**	5.50	9,20
**PYR**	6.80	9.14

FLU and ANT present a smaller width than PHE, permitting their inclusion in the cavity, but the distance between their molecules and the hydrophobic cavity of HP-β-CD is not as close as in the case of PHE, and the interaction is weaker. Similar results were obtained by Ko et al. [7] for the inclusion of naphthalene and PHE in HP-β-CD. For this reason E_deformation CD_ in these complexes is higher than for PHE complex, since the CD is deformed to improve the interaction with the PAHs (Table 4).

**Table 4:** Energy values (in kJ/mol) obtained through MM+ geometry optimization of PAH-CD complexes under study (suffixes a and b denote different starting orientations).

Complex	ΔE_complexation_	ΔE_interaction_	E_deformartionCD_
**ACE-HP-β-CD**	−72,54	−80,66	7,91
**ACE-RAMEB**	−95,52	−110,97	15,11
**ACE-HPγCD**	−89,53	−98,62	8,83
**ANT-HP-β-CD**	−69,65	−99,20	29,05
**ANT-RAMEB**	−95,73	−111,09	14,90
**ANT-HPγCD**	−104,52	−115,24	10,51
**FLT-HP-β-CD_a_**	−84,09	−118,83	34,37
**FLT-HP-β-CD_b_**	−79,74	−95,19	15,11
**FLT-RAMEB_a_**	−101,17	−118,96	17,20
**FLT-RAMEB_b_**	−110,21	−124,19	13,10
**FLT-HPγCD_a_**	−105,57	−117,66	11,76
**FLT-HPγCD_b_**	−106,82	−121,01	13,90
**FLU-HP-β-CD**	−69,02	−96,44	26,41
**FLU-RAMEB**	−97,49	−111,80	14,06
**FLU-HPγCD**	−97,86	−107,41	9,25
**PHE-HP-β-CD**	−81,37	−84,05	2,09
**PHE-RAMEB**	−83,38	−115,24	31,56
**PHE-HPγCD**	−107,41	−119,04	11,22
**PYR-HP-β-CD_a_**	−83,09	−89,12	5,53
**PYR-HP-β-CD_b_**	−86,65	−87,19	5,94
**PYR-RAMEB_a_**	−106,07	−122,77	16,24
**PYR-RAMEB_b_**	−105,36	−122,31	16,37
**PYR-HPγCD_a_**	−103,60	−117,58	13,48
**PYR-HPγCD_b_**	−109,96	−123,19	12,68

**Figure 2:**
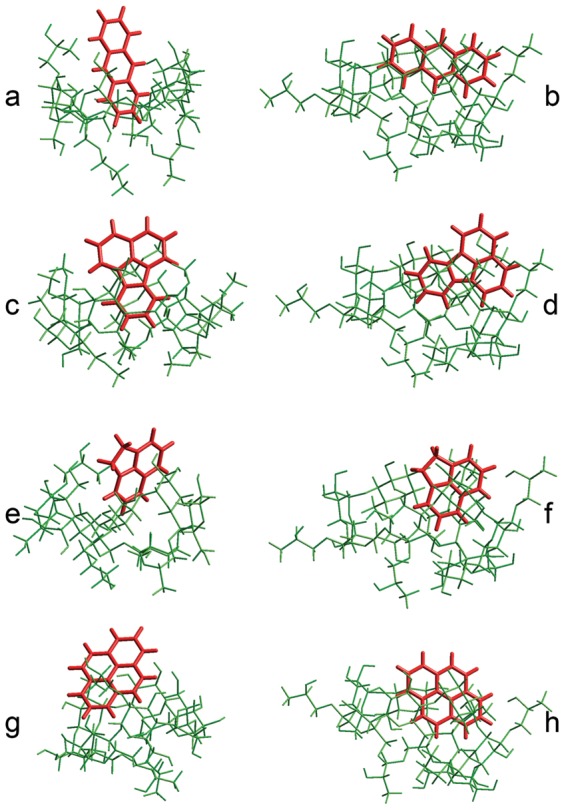
Structures of PAH-CDs complexes after their energetic minimization. ANT-HP-β-CD (a), ANT- HP-γ-CD (b), FLT- HP-β-CD (c), FLT-HP-γ-CD (d), ACE-HP-β-CD (e), ACE-HP-γ-CD (f), PYR- HP-β-CD (g), PYR-HP-γ-CD (h).

PHE, FLU and ANT molecules present a length higher than that of the internal cavity of HP-β-CD, and these compounds are included only partially in such cavity, remaining a relatively small part of their molecules into contact with water. As an example, the complex between ANT and HP-β-CD is presented in Figure 2a. This partial inclusion is more evident in the cases of PAHs with a width bigger than that of the CD cavity, such as FLT (fig. 2c), ACE (fig. 2e) and PYR (fig. 2g). In these cases, a great part of the PAH molecule is into contact with water, reducing their aqueous solubility due to having a great non-polar extreme in their inclusion complexes.

Due to the fact that one of the extremes of FLT is narrower than the other, the interaction with HP-β-CD occurs through two different binding modes (a and b). ΔE_interaction_ is more favoured in the first case (Table 4), since the molecule is more included into the hydrophobic CD cavity, and a closer contact with HP-β-CD cavity are taking place thanks to the higher deformation that the CD molecule can adopt (Table 4).

The comparative study of Kcs obtained in the case of using RAMEB shows that the complex ANT-RAMEB presented the highest value, about 10-fold higher than the lowest one, presented by PYR. The sequence of Se is in total agreement with that of Kc (Table 2), being remarkable the increase of ANT solubility more than 500-fold higher (Table 1), although those of PHE, FLT and FLU were also very high. ACE and PYR are again those that present lower Se (65 and 48-fold, respectively), although they are also important increments. RAMEB is a derivative of β-CD and, as discussed before, ANT and PHE are two of the six PAHs studied which better were accommodated to the dimensions of β-CD, although the sequence of their Kc and Se values in RAMEB was the contrary to that observed in HP-β-CD. Although ANT and PHE present lengths (9.14 and 9.20 Å, respectively, Table 3) bigger than that of β-CD (about 7.9 Å), the fact of its high solubility increase indicates that the methyl groups of RAMEB and their arrangement permit a closer interaction with both PAHs. According to Immel and Lichtenthaler [39], the introduction of methyl groups to O6H hydroxyl groups increases the depth of the cavity up to 10–11 Å, although the diameter of the cavity does not change. It indicates that probably only a very small part of both PAHs remains into contact with water, increasing their aqueous solubility. The same occurs in the case of FLU. In conclusion, the more appropriate lengths of PAHs in relation to that of RAMEB is probably the reason why Kc values obtained for complexes of ANT, PHE and FLU are higher than those obtained with HP-β-CD.

As in the case of the complexes with HP-β-CD, ACE and PYR presented the lower values of Kc and Se due to their bigger width in comparison to that of RAMEB cavity. However, in the case of the complex with FLT, in spite of having higher width than the cavity of β-CD derivatives, the solubility of the inclusion complex increased almost 400-fold higher than that of the free molecule, in comparison to those of ACE and PYR (65- and 48-fold higher, respectively), being even greater than that of FLU (a similar behaviour as that observed with HP-β-CD).

The comparative study of Kc and Se values corresponding to the complexes with HP-γ-CD showed an opposite sequence than in the cases of HP-β-CD and RAMEB, since the highest values were presented by PYR, followed by ACE. The derivatives of γ-CD show a cavity internal diameter about 7.5–8.3 Å and a length of 7.9 Å [35], therefore the accommodation of PYR molecule in this cavity was the best of all the PAHs studied, since its dimensions are quite appropriate (6.8 Å width, 9.14 Å length). The Kc for the complex PYR-HP-γ-CD is 1838 M^−1^, higher than those reported previously for the natural γ-CD, i.e., about 250–300 M^−1^ [40], [41], indicating a more favourable complexation with the HP-γ-CD derivative. In the case of ANT, its Kc obtained previously with γ-CD (240–330 M^−1^) [40], [42] were very similar to that obtained with HP-γ-CD in this work (234 M^−1^). Figure 2h shows a deeper inclusion of PYR molecule in HP-γ-CD in comparison to HP-β-CD (Figure 2g).

The inclusion of ACE in HP-γ-CD cavity is also slightly favoured in comparison to the β-CD derivatives due to its dimensions (5.89 Å width, 6.74 Å length, Table 3). Its width is so similar to β-CD cavity diameter that it is difficult to be completely included, as it can be observed in Figure 2e, but the contrary occurs in the cavity of HP-γ-CD (Figure 2f), and the interaction with its hydrophobic cavity is closer and stronger than that of ANT (fig. 2b), FLU, or PHE, which present smaller widths than that of ACE (4.91, 5.23 and 5.50 Å, respectively).

FLT also presents a width of 6.71 Å and length of 8.52, and its complex with HP-γ-CD showed also a high Kc value (701 M^−1^), very similar to that of ACE. It is due to the different orientation of FLT molecule when forming complex with HP-γ-CD, whose wider cavity permits the inclusion of FLT with its longer part perpendicular to the CD cavity (Figure 2d). However, Kc value of this complex is much lower than those obtained for their HP-β-CD and RAMEB complexes, due to the closer interaction between FLT molecule and the β-CD derivatives than with the wider cavity of HP-γ-CD.

Table 4 summarizes the results of geometry optimization processes for the different inclusion complexes. In all cases ΔE_complexation_ and ΔE_interaction_ are negative, indicating energetically favoured complexation processes. Nevertheless, these energy values are not clearly correlated with the experimental Kc ones for all the PAHs studied. The lack of agreement between the sequence of Kc and ΔE_complexation_ can be evaluated considering the presence of other factors. Considering the molecular properties of PAHs, the absence of ionisable groups or the impossibility to form hydrogen bonds limits the interaction after complexation to hydrophobic and van der Waals ones, which are the main driving forces [43], [44]. Respect to the hydrophobic interaction, our experimental design limits the correct evaluation of this factor, because in our simulations, performed under vacuum, does not exist a transfer of a hydrophobic guest (PAH) from an aqueous to a nonpolar environment such as the CD cavity [45]. Neither desolvation of hydrophobic guest nor the water molecules expelled from the CD cavity are taken in consideration. These phenomena can be influenced by the partial or total inclusion of guest into the CD cavity, but these facts are not reflected by the MM calculations [46]. According to Dodziuk [26] and Jaime et al. [47], the main consequence is that calculated energies only reflect enthalpy changes without the entropic contribution, making energy values at least as qualitatively useful. Nevertheless, the influence of water could be relativized because CD derivatives have a limited amount of water compared with natural CDs [48]. In addition, other authors [49] minimize the influence of water molecules expelled during complexation. According to these authors, due to enthalpy-entropy compensation, release of conformational strain and exclusion of cavity-bound high-energy water molecules do not contribute energetically to the complex, but these changes take place after complexation to maximize the contact between host and guest and establish stronger hydrophobic and van der Waals interactions.

**Figure 3:**
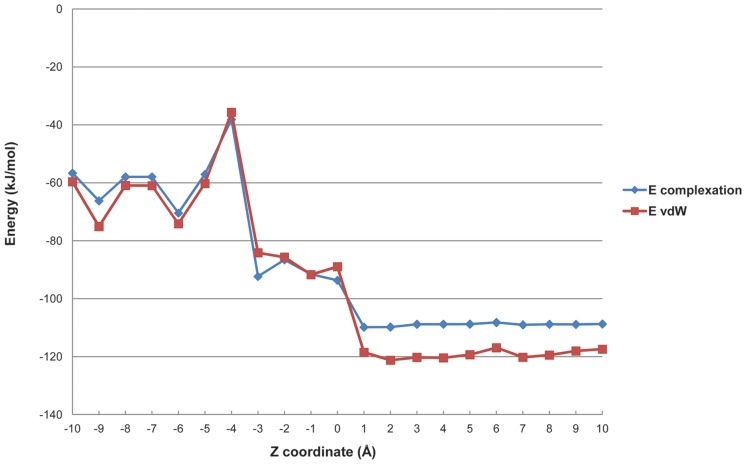
Complexation energies of PYR-HP-γ-CD inclusion complex. E_complexation_ and van der Waals energies (E_vdW_) (in kJ/mol) at different Z positions (Å), calculated by MM+.

Variations of E_complexation_ and van der Waals energies against the distance during the docking experiences are correlated in the vast majority of cases (data not shown), confirming the van der Waals interactions as the main driving force of complexation between PAH and CDs under our experimental conditions. As an example, Figure 3 shows that in the case of PYR-HP-γ-CD trends of complexation energy and van der Waals energy curves versus Z distance are similar, indicating that complexation is governed by van der Waals interaction. The energy variation involved in the inclusion process indicates that the inclusion complexes adopt geometry with the guest inside the cavity in order to increase the Van der Waals interaction.

E_deformationCD_ is a measure of conformational strain changes of CD after complexation. The main changes for this parameter were observed for HP-β-CD and RAMEB complexes (Table 4), in order to accommodate the PAH molecule into the CD cavity in those cases when penetration was effective. But considerable variations were registered also for HP-γ-CD complexes, favoured by the greater flexibility of the 8-glucopyranose ring of γ-CD compared with β-CD ones [9], in order to permit a closer contact between PAHs and HP-γ-CD.

Summarizing, the comparative study of the phase solubility diagrams of each PAH in the presence of the various CDs studied (in total 18 complexes) allows predicting which of them would be the best for reaching the highest solubilization of a specific PAH or group of PAHs among those studied here. PHE, ANT, FLU and FLT clearly presented their higher solubility when β-CD derivatives (HP-β-CD and RAMEB) were used, but the complexes with RAMEB were favoured, increasing their solubility up to 572, 41, 574 and 101 mg l^−1^, respectively. This is a consequence of the methylation of β-CD, which expands the hydrophobic cavity from the former 8 Å of the natural CD to 10–11 Å [39], thus allowing extra interaction for those molecules able to penetrate along the whole torus height. In the case of HP-β-CD, the presence of hydroxypropyl substituents may be related with steric hindrance to the penetration of guest into the CD cavity [50], [51], limiting in some extent the complexation process. This was reflected in the higher binding constants Kc observed with RAMEB compared with the hydroxypropyl derivative. On the contrary, PYR presented the best solubility results when using HP-γ-CD, but for ACE the use of any of the three CDs gave the same results, reaching a maximum solubility about 125–147 mg l^−1^.

It is interesting to emphasize also the change in the sequence of the apparent solubility among the different PAHs studied after their complexation with the different CDs with respect to their aqueous solubility (Table 2), as a consequence of the different solubilization efficiency (Se) obtained with each of the CDs used. These synthetic derivatives seem to have an equalizing effect: the solubility of the least soluble compounds was improved to a higher extent than that of the more soluble compounds. A similar trend was also observed by Balogh et al. [8] when studied the solubility of BTEX compounds using some derivatives of β-CD. This result could be an advantage in the application of these CD derivatives in soil remediation, since it would allow extracting a high percentage of those more persistent PAHs, increasing their bioavailability at the same time when the less persistent ones are also extracted.

In spite of these advantages of using CDs for soil decontamination, and many others such as the fact that they are environment-friendly compounds and biodegradable, the feasibility of their use concerning the price of bulk material is frequently argued for not selecting them as remediation technology. However, Gruiz et al. [52], who developed a comprehensive and uniform remediation technology-verification system for a comparative evaluation of using RAMEB with various realistic alternative technologies, demonstrated that the cost-efficiency was similar or even lower than in the others technologies, proving their efficiency and competitiveness. One of the strengths is that CDs technologies are less time consuming than other alternative ones (40–70% lower than the other treatments), and it compensates the price of the bulk material. It has to be taken into account that the price of HP-β-CD is even lower than that of RAMEB; however, that of HP-γ-CD is yet relatively high and has to be decreased for its use as additive in remediation technologies.
